# Epidemiology and clinico-pathological characteristics of current goat pox outbreak in North Vietnam

**DOI:** 10.1186/s12917-020-02345-z

**Published:** 2020-05-06

**Authors:** Trang Hong Pham, Mohd Azmi Mohd Lila, Nor Yasmin Abd. Rahaman, Huong Lan Thi Lai, Lan Thi Nguyen, Khien Van Do, Mustapha M. Noordin

**Affiliations:** 1 Faculty of Veterinary Medicine, Universiti Putra Malaysia, 43400 Serdang, Selangor Malaysia; 2 Faculty of Veterinary Medicine, Vietnam National University of Agriculture, Gia-Lam District, Hanoi, 010000 Vietnam; 3 Institute of Veterinary Research and Development of Central Vietnam, Nha Trang, Khanh Hoa 650000 Vietnam

**Keywords:** North Vietnam, Goat pox, Epidemiology, Pathology, Industry

## Abstract

**Background:**

In view of the current swine fever outbreak and the government aspiration to increase the goat population, a need arises to control and prevent outbreaks of goat pox. Despite North Vietnam facing sporadic cases of goat pox, this most recent outbreak had the highest recorded morbidity, mortality and case fatality rate. Thus, owing to the likelihood of a widespread recurrence of goat pox infection, an analysis of that outbreak was done based on selected signalment, management and disease pattern (signs and pathology) parameters. This includes examination of animals, inspection of facilities, tissue sampling and analysis for confirmation of goatpox along with questionaires.

**Results:**

It was found that the susceptible age group were between 3 and 6 months old kids while higher infection rate occurred in those under the free-range rearing system. The clinical signs of pyrexia, anorexia, nasal discharge and lesions of pocks were not restricted to the skin but have extended into the lung and intestine. The pathogen had been confirmed in positive cases via PCR as goat pox with prevalence of 79.69%.

**Conclusions:**

The epidemiology of the current goat pox outbreak in North Vietnam denotes a significant prevalence which may affect the industry. This signals the importance of identifying the salient clinical signs and post mortem lesions of goat pox at the field level in order to achieve an effective control of the disease.

## Background

The re-emerging of Capripoxvirus and it’s clinical syndrome has been well documented worldwide especially in Asia and Africa [1, 2]. Undoubtedly, this virus bears pronounced economic impact not only to endemic regions especially to the livelihoods of small-scale farmers and poor rural communities [3] but also posed major constraint in international livestock trade. A greater concern is the risk of its expansion to many countries including Vietnam in 2005 [4] which is in the midst of developing a competitive goat industry. The first reported goat pox outbreak of North Vietnam in 2005 that affected four provinces i.e. Coa Bang, Bac Giang, Lang Son and Ha Tay has led to the death of 789 goats. The agents confirmed via ELISA and PCR yielded that the isolate was host specific being severe in goats [4]. Following this incidence, the outbreak has been resolved leading to an annual increase of 38% in Vietnam goat population from 1.8 million heads in 2015 to 2.6 million heads in 2017 [5]. Owing to the Vietnamese government aspiration to produce 3.9 million heads of goats in 2020, a much more comprehensive study on devastating disease like the epidemio-economical impact of goat pox is warranted. Nevertheless, despite the increase in goat population in addition to animal movement along the borders, market demands, high stocking densities and proximity of facility, goat pox outbreak has recurred commencing from 2014 in Ninh Binh province. This recurrence has raised concern on the possible devastating impact of goat pox on Vietnam’s goat industry which forms the basis of this study. A thorough analysis of current recurrence along with a complete set of epidemiological data will confer an effective control and prevention of new outbreaks.

## Results

### Observation of the farms

Vaccination against goat pox was not practised in either type of farming systems. The main goat rearing methods in North Vietnam under the extensive system includes backyard farming where the goats are allowed to freely graze in lowland and mountainous areas. Under such system there is minimal provision of commercial feed. On the otherhand, under intensive farming, the goats are kept in stalls and supplemented with concentrates.

#### Morbidity rate

The morbidity and mortality rates due to goat pox is shown in Table 1. During this study, the first case of sick goats was reported in Ninh Binh province which then radiated to other parts of North Vietnam. Thus, the study commenced in Ninh Binh and radiated out to its five other surrounding provinces. The morbidity rate ranged between 11.8–17.5% without significant differences between all provinces except for Yen Bai which has the lowest rate (*p* < 0.000). However, this lowest rate at Yen Bai was not significantly different to that seen in Hoa Binh.

**Table 1:** Morbidity rate of goatpox outbreak in North Vietnam

Province	n	Morbidity (%)	*p-value*	OR	95% CI	
Bac Giang	2350	17.5 ^a^	0.000	1	–	–
Ha Noi	1295	15.7 ^a^		0.87	0.72	1.04
Hoa Binh	1954	13.9 ^a,b^		0.76	0.64	0.90
Nghe An	1798	17.1 ^a^		0.97	0.83	1.14
Ninh Binh	2435	16.5 ^a^		0.92	0.79	1.07
Yen Bai	1856	11.8 ^b^		0.63	0.53	0.75

*OR* odds ratio, *95% CI* confidence interval

Values within column bearing similar superscript do not differ at *p* < 0.000

### Mortality and case fatality rate of goat pox outbreak

Table 2 shows the case fatality rate of goats due to the infection during the study period. The mortality and case fatality rate ranges between 5.1–7.4% and 35.3–63%, respectively without any significant differences between provinces.

**Table 2:** Mortality and case fatality rate of goat pox disease in North Vietnam

Province	No. of affected goat	No. of death	Mortality (%)	Case fatality rate (%)
Bac Giang	411	145	6.17	35.28
Ha Noi	203	74	5.17	36.45
Hoa Binh	227	125	6.40	45.95
Ninh Binh	401	151	6.20	37.66
Nghe An	308	131	7.28	42.53
Yen Bai	219	138	7.44	63.01
Grand Total	1814	764	6.54	42.12

### Infection rate between farming system

It was found that goats under the extensive system has a 8.7% higher (*p* < 0.05) infection rate than those managed intensively (Table 3).

**Table 3:** Comparison of goat pox incidence based on rearing method

Method	n	Unaffected (%)	Affected (%)	*p-value*	OR	95% CI	
Extensive	4729	83.7	16.3	0.040	1	–	–
Intensive	6959	85.0	15.0		0.90	0.81	1.00

*OR* odds ratio, *95% CI* confidence interval

### Age susceptibility

In order to examine the influence of age to infection rate, the goats were into categorized into three groups, viz.; less than 3; 3–6 months and more than 6 months old. The analysis of age susceptibility to infection is shown in Table 4. It was found that at almost all instances, those between the ages of 3–6 months were most susceptible (*p* < 0.001) except at Ninh Bin province. The other age groups of less than 3 and more than 6 months have comparable infection rate.

**Table 4:** The infection rate based on age groups

Province	Total	< 3 months			3–6 months			> 6 months			Chisq	*p*-value
		n	Affected	Infection rate (%)	n	Affected	Infection rate (%)	Nn	Affected	Infection rate (%)		
Bac Giang	2350	545	86	15.8 ^a^	608	167	27.5 ^b^	1197	158	13.2 ^a^	58.31	0.000
Ha Noi	1295	315	45	14.3 ^a^	305	72	23.6 ^b^	675	86	12.7 ^a^	19.37	0.000
Hoa Binh	1954	528	64	12.1 ^a^	352	91	25.8 ^b^	1074	117	10.9 ^a^	51.46	0.000
Ninh Binh	2435	587	93	15.8 ^a^	623	173	27.8 ^b^	1225	135	11.0 ^a^	84.43	0.000
Nghe An	1798	404	63	15.6 ^a^	441	119	27.0 ^b^	953	126	13.2 ^a^	41.09	0.000
Yen Bai	1856	419	41	9.8 ^a^	492	98	19.9 ^a^	945	80	8.5 ^a^	42.89	0.000
Total	11,688	2798	392		2821	720		6069	702			

Values between columns bearing similar superscript do not differ at *p* < 0.000

*n* number of animals

### Clinical and pathology findings

**Fig. 1:**
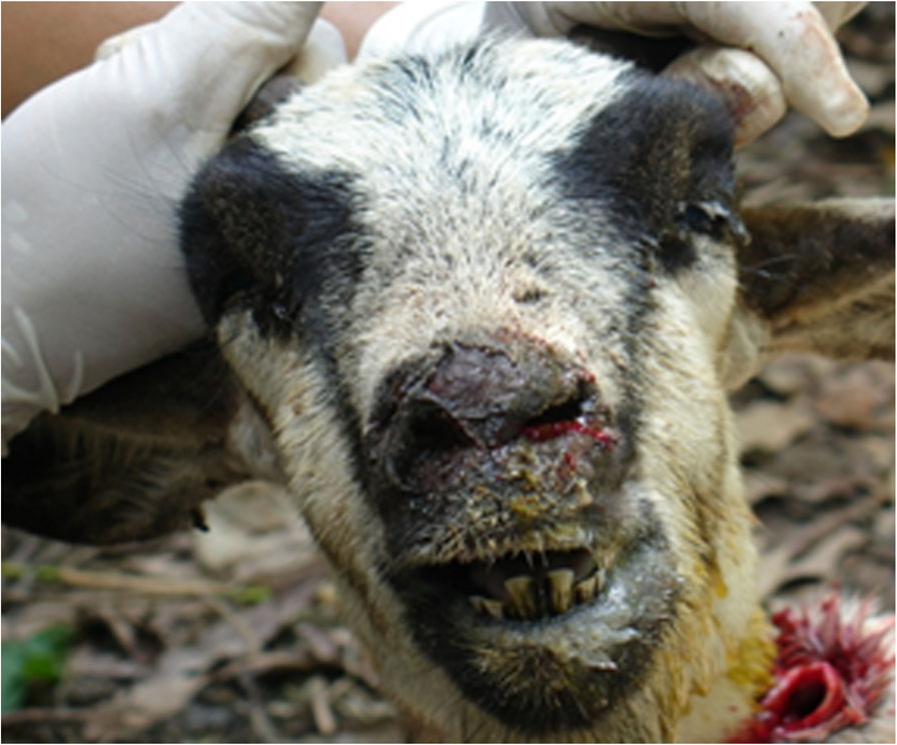
Photograph showing ulcers in nasal cavity and rhinitis

Goats showed varying degrees of clinical signs severity, however, almost 85% of infected goats showed loss of appetite, anorexia to completely refusal of feed leading to emaciation (Table 5). Fatigue and pyrexia were also among common manifestations observed in most cases. Additionally, blepharitis, rhinitis (Fig. 1) and difficulty to move ensued in some cases.

**Table 5:** Distribution of clinical signs of goat pox based on their occurrence (*n* = 1814)

Clinical signs	n	%
Loosing weight	1542	85.01
Anorexia	1542	85.01
Blepharitis	1450	79.93
Rhinitis	1134	62.51
Papules on skin	1269	69.96
Labor breathing	816	44.98
Pyrexia (> 40 °C)	634	34.95
Necrotic skin	408	22.49
Refusing feed	363	20.01
Difficulty to move	181	9.98
Blindness	45	2.48

**Fig. 2:**
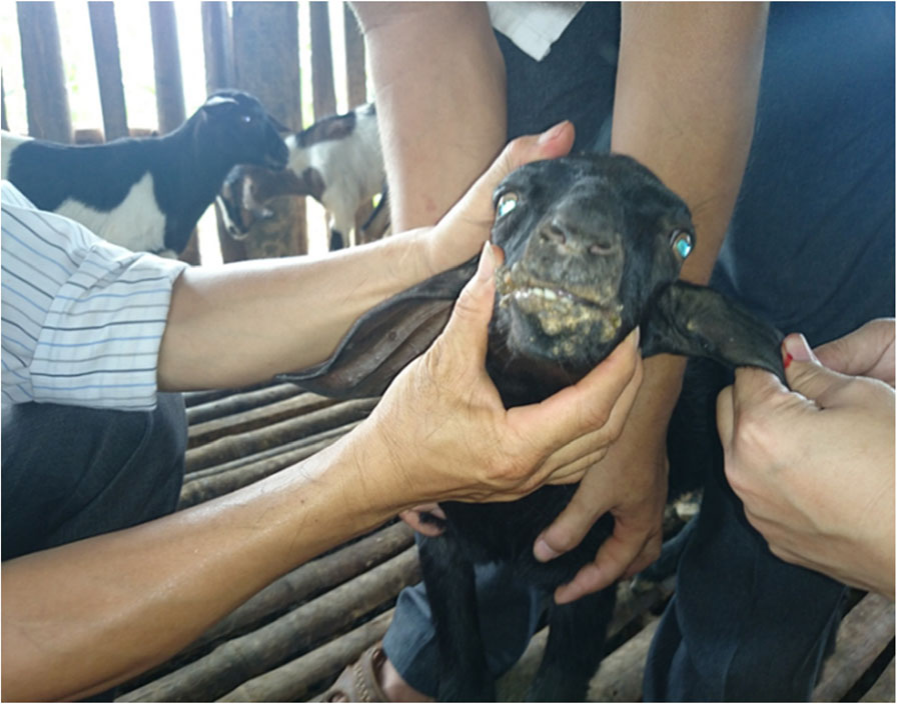
Photograph exhibiting papules found on mouth, nares and ear

**Fig. 3:**
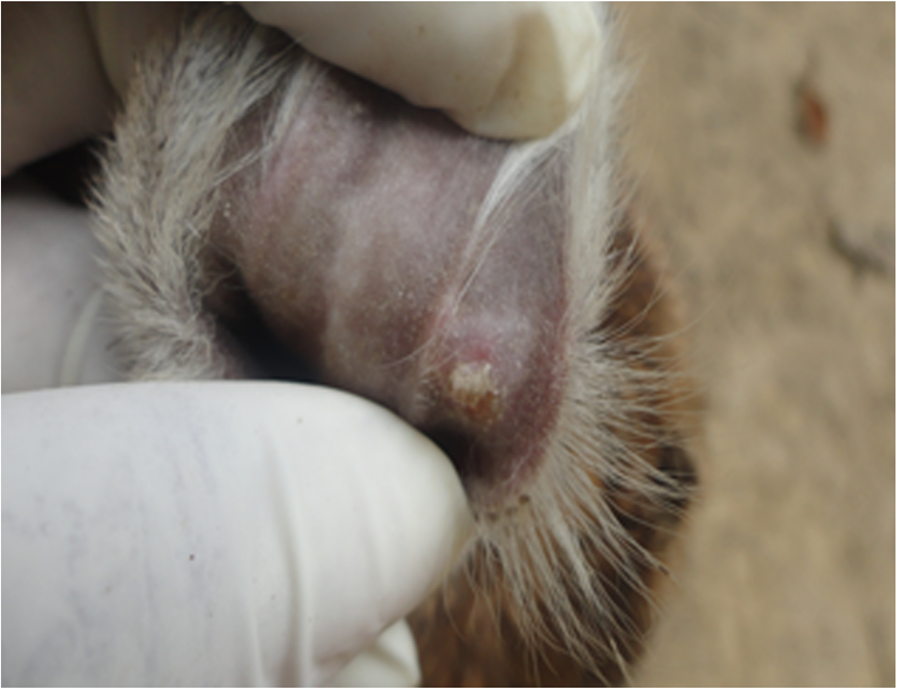
Photograph showing a papule that has ulcerated on the ear pinna

**Fig. 4:**
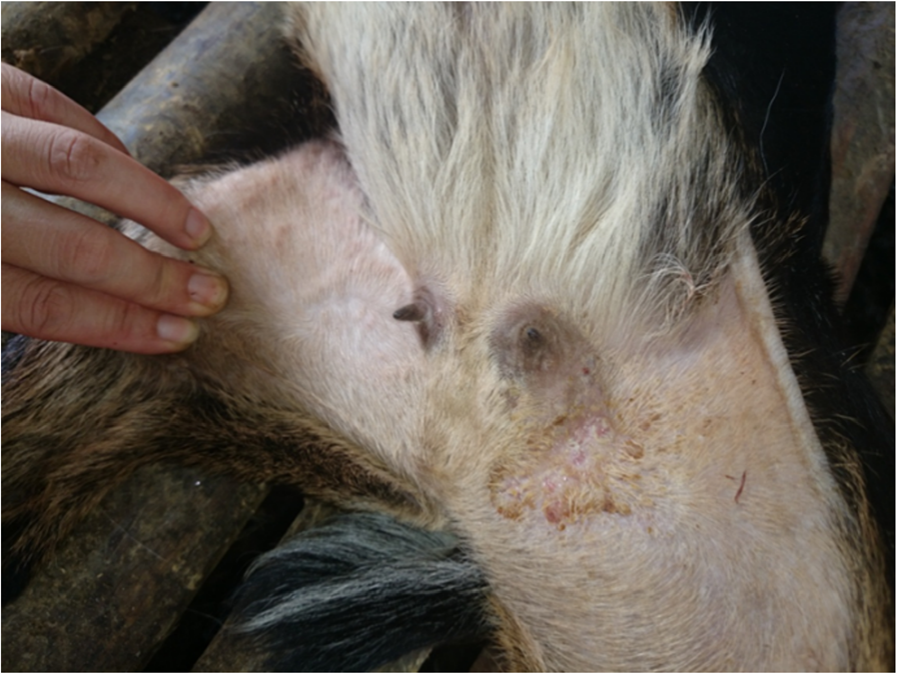
Photograph of infected goat’s udder denoting ulcers and inflammation

Hardened swelling which developed into sores were found on the skin (mainly hairless regions) over any part of the body including the mouth, pinna (Fig. 2) and udder (Figs. 3 and 4). The size of the pock lesions varies between 0.5–1 cm in diameter.

**Fig. 5:**
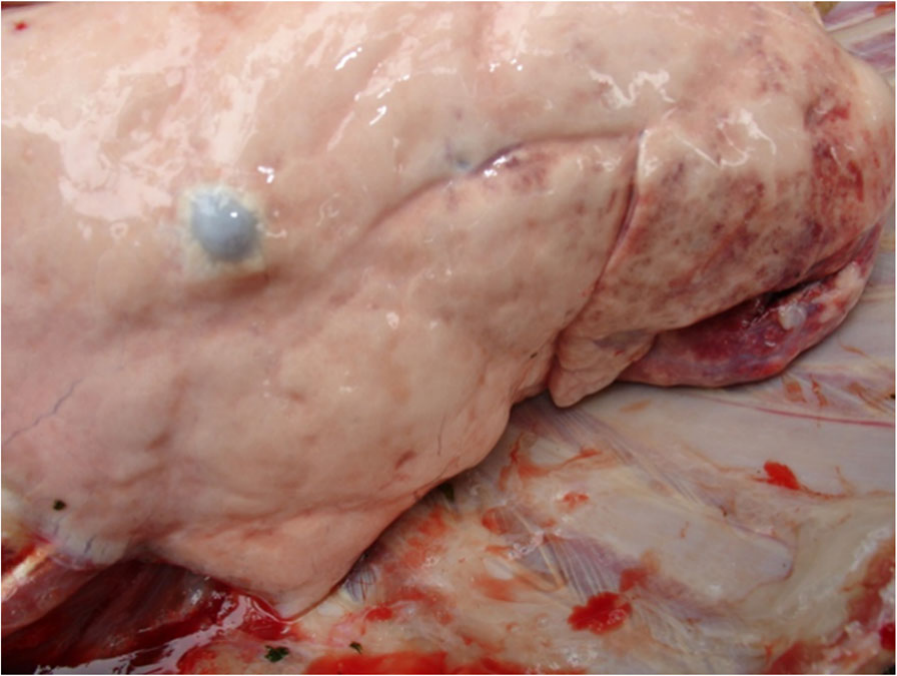
Photograph of a well-circumscribed greyish pock lesion in the lung of an infected goat

**Fig. 6:**
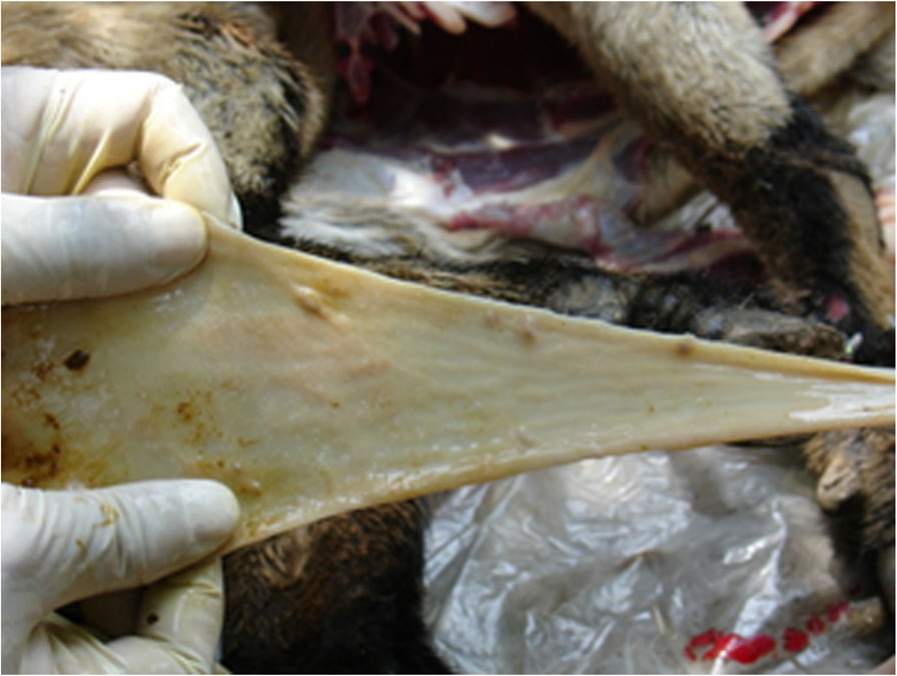
Photograph of calcified papules on the intestinal mucosa of an affected goat

The finding of lesions ante- and post mortem is presented in Table 6. In live animals, majority of lesions are confined to the eyes, nares and skin while that of post mortem revealed the lungs (Fig. 5) as a primary site. Calcified greyish papules were found in the intestines (Fig. 6), urinary bladder and uterus. However, other less frequently sites and tissues were also affected as shown in Table 6.

**Table 6:** Lesion distribution in selected organs and their frequency of appearance (*n* = 128)

Organ	Lesion	n	%
Eyes	Hyperaemia, corneal opacity, papules on the eyelids	128	100
Nares	Thickened and ulcerated papules on the nasum	128	100
Skin	Papules scattered all over the body	128	100
Trachea	Blood/fluid-filled vesicles	85	66.4
Lung	Inflammation and necrosis	128	100
Heart	Pale and flabby	52	40.6
Intestine	Calcified papules	64	50
Bladder	Papule	28	21.9
Uterus	Papule	22	17.2
Mesenteric Lymph Node	Necrosis	88	68.8

**Fig. 7:**
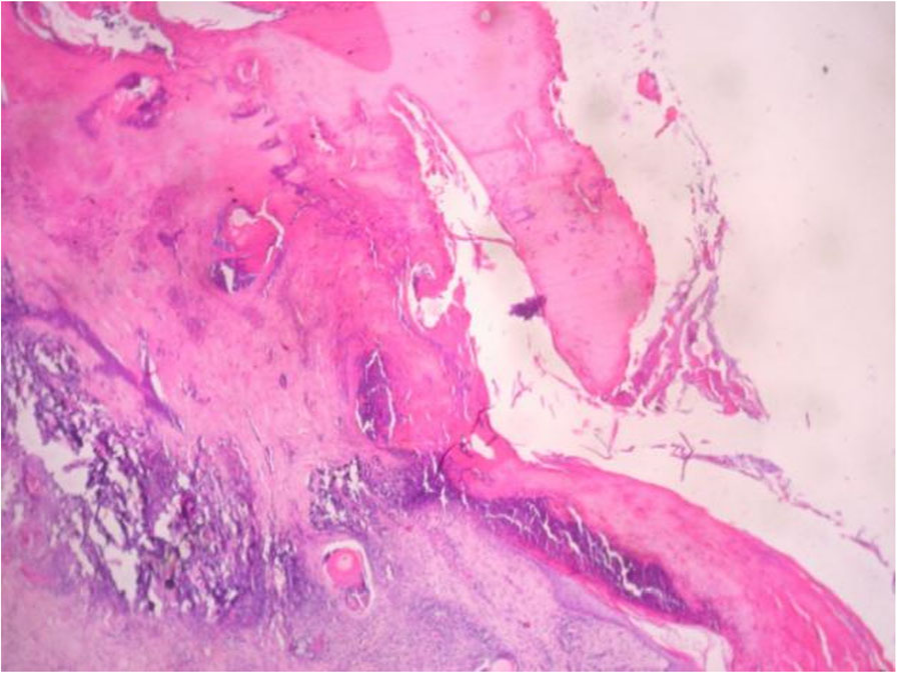
Damaged epithelial layers of skin of an infected goat (H&E, X10)

Histopathological lesions comprising of cellular degeneration and necrosis along with inflammation and haemorrhage were mostly found in skin (Fig. 7), lung and liver. Despite exhaustive histopathology search, no evidence of eosinophilic inclusions were seen in any tissues.

### PCR

**Fig. 8:**
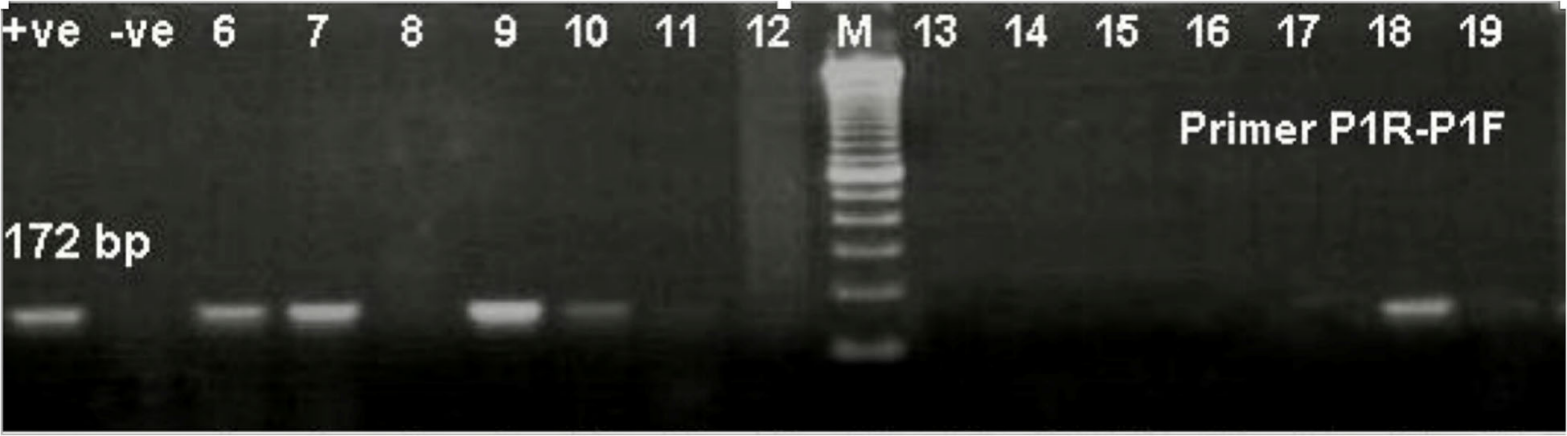
PCRA gene based PCR result for detection of capripox virus. Lane M: 100 bp ladder

The PCR primer specific test was performed on 128 scab biopsy samples. A total of 79.6% (102/128) of the samples were positive to capripox virus within the expected size band of 172 bp (Fig. 8).

## Discussion

Reported outbreaks of goat pox worldwide yields differing mortality rates with 7% in Sudan [2], 21% in Iraq [6] and 30% in India [7]. In this study, a much lower mortality rate was found despite a rather high morbidity rate high probably as a result of the study population containing comparatively fewer of the 3–6 months old goats. It has been shown that maternal antibody for goat pox is maintained for about 3 months and those animals older than 6 months that survived an infection will have life-long immunity [8, 9]. This phenomenon explains the susceptibility of those in the 3–6 months old [9] which should yield higher morbidity rate. However, since the number of animals under this group is quite low, the mortality rate has failed to surpass those of other groups.

The number of dead animals during the outbreak depends on the virus virulence, size of the population and their susceptibility and on the basic reproductive number i.e., average expected number of secondary cases produced by a single infection in a completely susceptible population [10]. However, these rates may vary depending on additional factors including breed [11] and the most notably the herd immune status [12]. Recently published data showed that case fatality rate of goatpox disease ranged from 21.4 to 60% [13–15]. Likewise, the high fatality rate in the present study underlined the need for a much more effective control of goat pox along with the requirement to vaccinate susceptible herd or in endemic areas. However, the difficulty in implementing such health programs in Vietnam is explained below.

A 23% morbidity rate based on seroprevalence has been documented in nomadic goat herds in Punjab [16]. It is not suprising to see a higher infection rate in the extensive system as previously reported [11]. However, this rearing method is popular with poor farmers in lowland and mountainous areas in Vietnam who could not afford to spend on a standard health management. Goats under the extensive system forage freely in a wide area exposing them increase chances to be exposed to the virus. These goats might have also been exposed to lesser domestication, maintaining many of the behavioural traits of the wild types such as aggressiveness [17, 18]. Furthermore, goats especially under the extensive system being naturally aggressive [17] predisposes the body to injuries making easier access of the virus when inoculated. This is an added problem since most of the goats were not dehorned (due to financial constraints) making injuries prone to infection during a fight. On the contrary, the low infection rate under the intensive system could have resulted from a much more efficient disease control program that has minimized spread of the virus within the herd. However, the benefits of extensive farming system can be still exploited by taking advantage of its eco-agrarian nature. It can economically ultilise marginal or unused land that can be later be easily adapted by the goats. Such conditions had less stressful effect on the goats making them much more hardy to harsh conditions. This is an opportunity for the poor rural farmers with limited financial resources and knowledge in commercial goat farming. This can be improved if there is provision of extension veterinary officers to offer guide and assistance in goat farming.

Undoubtedly, defining the vulnerable period of infection is one of the most important measurement to be known for an effective disease management [19]. In the study presented here, the most susceptible age were goats of 3–6 months old which conforms to findings of [16, 20] who found that the chance of infection chance in the young was 2.2 times greater than that of an adult. However, contradictory results were seen if infection rate was based on seroprevalence. Fentie et al. [20] demonstrated a low infection rate in older animals although this appeared to refute earlier published findings [21]. Nevertheless, in the latter study [21], age groups were not clearly defined which may have led to a less homogenous groupings. Additionally, the collected samples from slaughter house, tanneries and hide markets where probable that few samples were collected from goat kids to be devoid [21]. The age grouping the study presented here was based on the main purpose of meat goat breeding in Vietnam. The indigenous and mixed breed of Vietnamese goat attained a market weight of 25 to 30 kg at 6 months old age, justifying a 3 month interval being chosen.

Recognising the key salient clinical signs is key factor for field diagnosis of goat pox [11]. The prominent clinical signs seen in this study too were depression and being much more severe in kids [22, 23] accounting for systemic signs of pyrexia. About 85.01% of affected animals showed varying degrees of anorexia associated with the development of lesions on mucus membrane of the face. The lesion commences as red patches around the mouth, nose and eyes which later swelling into a papule. These papules trigger lacrimal, nasal and saliva discharges. Respiratory distress and secondary bacterial pneumonia are predominant in kids which could not survival malignant stage [6, 24, 25]. In adult goats, the ulceration of papules renders difficulty for digestive and breathing activities which in turn worsen productive performance. The goats with conjunctivitis, corneal opacity and blepharitis emulated the acute phase pox disease [4]. The development of pox lesions is observed over the animal body especially hairless areas (face, pinna of the ears, udder, genital, anus, under the tail). The red patches turn to hard rubbery papules and become vesicles after 3 to 4 days. Necrotic papules formed pustular as the result of thrombosis and localised ischaemia. Dark hard scabs are formed by the remnant of necrotic papules [6, 25–27].

Although the overt clinical signs of goat pox are quite characteristic, the less severe manifestation needs to be judiciously distinguished from several other closely resembling diseases. The closest would be contagious ecthyma (orf) which affects young kids while goat pox involves all ages. The signs are usually that of flat or dome-shaped bullae crust around the commissures of mouth which left no scar after healing [28] as opposed to a rather permanent papular lesion in goat pox. Blue-tongue may be confused with goats pox although the goats are less less susceptible with signs rarely seen in goat pox i.e. localized oedema, haemorrhages and erosion of mucous membrane. The post mortem lesions of blue tongue are that of effusion in the thoracic cavity and pericardial sac [29]. High mortality is seen in peste des petits ruminants (PPR) which affects mainly young goats that showed signs of coughing; halitosis, erosive oral lesion and severe diarrhoea. These signs are not seen in goat pox along with rather pathognomonic lesion of PPR comprising of zebra stripes of gastro-intestinal tract and pneumonia [30]. Lastly, a likely differential to be considered to goat pox is dermatophilosis [31] where the latter exhibited signs of paintbrush matted hair all over the body that is not a feature of goat pox.

In this study, for all PCR positive cases, the clinical and post mortem lesions were 100% present in the skin and lungs of affected goats. It is likely that owing to the epitheliotropic nature of the virus lesions were predominantly seen in the skin, lung and discrete sites within mucosal surfaces of oro-nasal and gastrointestinal tissues [4]. As evidenced in this study and as reported earlier in similar studies, the role of skin and lung as a target organ [32] for the virus leads to much more deposition of the lesion in these tissues [33, 34]. Beside darkened circumscribed pox lesions [33, 35], the entire lung are pale pink with loss of sponginess. Congested trachea contain blood or fluid-filled vesicles with involvement of the lymph nodes. As seen in the study presented here, calcified nodules are found the most abudant in large intestine (rectum) of goats that were mildly affected [21, 36].

Histopathological findings in the study presented here were in accord to previous publications registering marked change in the epidermis. The degeneration of epithelial cells, hyperkeratosis, ballooning and degeneration of proliferating epithelial cells along with inflammation led to the desquamation of skin layers. Variable observation of lung microscopy include haemorrhage, congestion and thickening alveoli wall which resulted in narrowed alveoli. Secondary bacterial infection has invoked infiltration of inflammatory cells to affected regions of the lung [6, 37, 38].

The PCR-based test is chosen because of its sensitivity and simplicity [39]. The sensitive and simple PCR assay has confirmed caprine pox virus in the biopsy samples [40]. Almost 80% of the samples were positive with amplicon size of 172 bp although no attempt was made to identify and differentiate of caprine pox virus [1, 22, 41, 42]. However, the isolates from this study did not show much variation compared to those reported in China [43]. This could be explained by the fact that although phylogenetically China has three main subgroups of goatpox, only one is circulating in the south i.e. bordering Vietnam [43].

These findings pose a challenge to the aspiration of Vietnam’s to transform the future potential of goat farming into an industry. The local consumer prefers fresh chevon than frozen products due to food safety issues linked to the weakness of their cold chain system [44]. Furthermore, goats as well as being a form of meat for the family and community, goat serves as a cash reserve for the poor farmer [45].

The current study also revealed most of the goat husbandry system is mainly extensive which may hamper the possibility to initiate goat production within the mountainous areas. Likewise, as revealed here, goats reared under the intensive system offers a better farming milieu for disease control which the farmer or nation should adopt to improve productivity. Under an intensive system, the ease to isolate and locate an infected animal and area enabling an effective diagnosis and thus control and prevention. It is rather difficult or almost impossible to perform such tasks (isolate and locate) under free grazing or nomadic conditions. Nevertheless, Vietnam should make formidable reforms to the livestock industry since goats in Vietnam are still (as found in this study) and in future will be reared by the poorer farmers halting an increase in goat population and productivity. This is even much more worrying especially with respect to a lack of herd health program (disease control). Thus, in order to bring the industry to greater heights, offsetting devastating disease like goat pox is mandatory. It is believed that these findings on goat pox will facilitate the government to continue working on improving disease identification and control to avoid hindrance in goat production.

## Conclusions

Goat pox infection in North Vietnam if left unattended may lead to devastating effect to the goat industry. Thus, needs arises not only to effectively control the disease but also to downregulate risks factors involved including that of current state of rearing. This includes provision of veterinary extension services to the poor farmers adopting the extensive system in order to improve productivity via an effective herd health program.

## Methods

### Ethics, consent, questionnaire and study area

Since North Vietnam does not impose ethics on the use of local animals for research, all procedures involving in this study were conducted in compliance to the recommendations of the Guide for the Care and Use of Agricultural Animals in Research and Teaching (2010) [46]*.* A well-defined questionaire composed of farm management information (total number of animals/age groups, breed, farming system and detailed health status) relevant to goat pox were noted during the visit and all participating farms consented the research via a written permission.

The sample size (n) was determined using the formula:

$$ \mathrm{n}={\mathrm{Z}}^2\mathrm{pq}/\mathrm{L} $$

where, Z = standard normal distribution at 95% confidence interval = 1.96

= prevalence of similar work (Babiuk 2008) = 33%

$$ \mathrm{q}=\mathrm{p}\hbox{-} 1 $$

$$ \mathrm{L}=\mathrm{allowable}\ \mathrm{error}\ \mathrm{taken}\ \mathrm{at}\ 5\%=0.05 $$

Thus, the minimum required sample size obtained from the formula for this study was 477.

Disease investigation had been conducted in six provinces in North Vietnam where goat farming is most actively conducted (Fig. 8). In general, goat farming in Vietnam is mainly divided into either extensive or intensive system as previously described [47]. During the visit, farms with clinically affected goats and those in close contact with the herd within outbreak provinces were further assessed. A thorough physical examination of clinical signs was done with emphasis on predilection site of goat pox lesions and animals with severe clinical signs were then post mortem.

### Questionaire and data collection

The questionnaire was structured to encompass information of the farm, management system practiced by the owner during an interview. It is compartmentalised to contain three main sections namely; ownership and farm information, herd information and physical plus pathology findings. The template of this questionnaire is attached separately as an Additional file 1.

### Tissue sampling

Based on the physical examination, a total of 11,688 goats that falls under the category of being affected or those in contact were chosen. Out of these, 1481 had clear cut signs suggestive of goat pox whereby fresh tissue samples totaling to 128 were collected for further pathology and virology diagnoses.

Approximately 2–3 g of lesions were taken and placed in PBS (7.2 pH with 1% gentamycin) and stored under chilled conditions during delivery. Samples were then transferred to Key Veterinary Biotechnology Laboratory, Vietnam National University of Agriculture, Hanoi, Vietnam. Roughly a 1 cm^3^ lesion the of skin, lung, heart, liver, intestine, spleen, kidney and lymph node were fixed in 10% buffered formalin and later processed using routinely for histopathological examination.

### Polymerase chain of reaction (PCR)

DNA extraction was performed using DNeasy Blood Tissue Kit (Qiagen, Gemany) following manufacturer instruction. Primers used for identifying Capripoxvirus in clinical specimens as previously designed [39].

The forward primer was P1: 5′-TTTCCTGATTTTTCTTACTAT-3 ‘and the reverse primer was P2: 5’-AAATTATATACGTAAATAAC-3′. 50 μl of reaction mixture contained 5 μl buffer, 3 μl of MgCl_2_, 2 μl of dNTP mix (10 mM), 2 μl (10 pmol/μl) of each primer, 0.4 μl of Taq-DNA, 12 μl biopsy supernatant and 23.6 μl of RNAse free water. PCR cycle started with initial denaturation at 94 °C for 5 mins, followed with 35 cycles (1 min each) of denaturation at 94 °C, annealing at 50 °C, extension at 72 °C and final extension at 72 °C for 10 mins. The PCR products were examined by 1.5% agarose gel electrophoresis with ethidium bromide staining.

### Data analysis

All data obtained was subjected to statistical analysis using the SAS 9.0 (2002), USA and only differences of *p* < 0.005 were considered as significant.

## Supplementary information

**Additional file 1.** Questionnaire.

## Data Availability

The datasets generated and/or used during the current study are not available to public as it is owned by the Vietnam National University of Agriculture, Vietnam. However, these can be requested via email from the corresponding authors; Dr. Pham Hong Trang (htrang2910@gmail.com) and/or Prof. Dr. Mustapha M Noordin (noordinmm@upm.edu.my).

## Abbreviations

bp: base pair
PBS: Phosphate buffered saline
PCR: Polymerase chain reaction
OD: Odds ratio
CI: Confidence interval
n: number of animals
Chisq: Chi Square test
