# Supplementary material for: Epidemiology and clinico-pathological characteristics of current goat pox outbreak in North Vietnam
Source: BMC Vet Res. 2020 May 6;16:128. doi: 10.1186/s12917-020-02345-z (PMC7203824; doi:10.1186/s12917-020-02345-z)
Supplement: Supplementary file 1 — Additional file 1. Questionnaire. [file 12917_2020_2345_MOESM1_ESM.docx]

**QUESTIONAIRE FOR EPIDEMIOLOGICAL STUDY**

| Vietnam National University of Agriculture  Faculty of Veterinary Medicine | **Socialist Republic of Vietnam**  **Independence – Freedom - Happiness** |
| --- | --- |

**EPIDEMIOLOGICAL SURVEY OF GOAT POX**

I. OWNERSHIP & FARM INFORMATION

1. Owner’s name: ……………………………………………………………………………………

2. Address: ……………………………………………………………………………………………

District: ………………………………………… Village: ………………………………………….

Province: ………………………………………………………………………………………………

3. Time of interview: …………………………………………………………………………

4. Season (tick √ in appropriate box):

| Spring  (Feb to April)  …………. | Summer  (May to Jul)  ………….. | Autumn  (Aug to Oct)  …………. | Winter  (Nov to Jan-following year)  ………………. |
| --- | --- | --- | --- |

II. HERD INFORMATION

1. Total No. of goat: ………………… head

2. Breed: ………………………………………………………………………………………………

3. Rearing method (tick √ in appropriate box):

| Extensive  …………… |  | Intensive farming  ……………… |
| --- | --- | --- |

4. Main source of food supply: …………………………………………………………………

……………………………………………………………………………………………………………..

…………………………………………………………………………………………………………....

5. No. of goat (age grouping)

| **Age group** | **Male** | **Female** | **Total** |
| --- | --- | --- | --- |
| Neonate - <3 months |  |  |  |
| 3 - <6 months |  |  |  |
| 6 - <12 months |  |  |  |
| > 12 months |  |  |  |
| **Grand Total** |  |  |  |

III. PHYSICAL EXAMINATION AND PATHOLOGY

6. Physical examination (tick √ the appropriate box) – for each goat

| **Item** | **Yes** | **No** |
| --- | --- | --- |
| Fatigue |  |  |
| Anorexia |  |  |
| Pyrexia |  |  |
| Red patches on skin |  |  |
| Vesicle forming papules |  |  |
| Pox lesions on face |  |  |
| Pox lesions on hairless areas (inguinal, udder, vulva, penis) |  |  |
| Swollen lymph nodes (submandibular, groin etc) |  |  |
| Lacrimal, nasal and/or salivary discharges |  |  |
| Pneumonia |  |  |
| Abortion |  |  |
| Swelling of the mammary gland/udder |  |  |

7. Post-mortem observation (tick √ the appropriate box) – for each suspected goat

| **Item** | **Yes** | **No** |
| --- | --- | --- |
| Hyperemia, haemorrhage, edema skin |  |  |
| Proliferative or enlarged lymph nodes |  |  |
| Papules on nose, mouth and ear |  |  |
| Ulcers on nares, gums and tongue |  |  |
| Pox lesion in the respiratory tract |  |  |
| Papules on vulva, udder/penis, testicles |  |  |

8. Summary of affected case record regarding to age

| **Age group** | **Positive** | |  |
| --- | --- | --- | --- |
|  | **Male** | **Female** | **Total** |
| New born to below 3 months |  |  |  |
| 3 to below 6 months |  |  |  |
| 6 to below 12 months |  |  |  |
| Above 12 months |  |  |  |
| **Total** |  |  |  |

9. Others clinical signs/lesions: ……………………………………………………….

…………………………………………………………………………………………………………….

……………………………………………………………………………………………………………

…………………………………………………………………………………………………………….

| Day ……….. Month ……….. Year ……….. | | |
| --- | --- | --- |
| Interviewer | Animal owner | Pathologists |
